# Cine-MRI for Quantifying Uterine Peristalsis: A Systematic Review and Meta-Analysis

**DOI:** 10.3390/jcm14031021

**Published:** 2025-02-06

**Authors:** Angela Vidal, Cristina Bora, Jeannette von Holzen, Marietta Gulz, Verena C. Obmann, Janna Pape, Tanya Karrer, Gürkan Yilmaz, Michael von Wolff

**Affiliations:** 1 Division of Gynecological Endocrinology and Reproductive Medicine, Women’s University Hospital, Inselspital Bern, University of Bern, 3010 Bern, Switzerland; 2 Fertisuisse, 4600 Olten, Switzerland; 3 Department of Gynecology and Obstetrics, Bern University Hospital, University of Bern, 3010 Bern, Switzerland; 4 Department of Diagnostic, Interventional and Pediatric Radiology, Inselspital, Bern University Hospital, University of Bern, 3010 Bern, Switzerland; 5 Department of Radiology, Zuger Kantonsspital, 6340 Baar, Switzerland; 6 Medical Library, University Library of Bern, University of Bern, 3010 Bern, Switzerland; 7 Centre Suisse d’Electronique et de Microtechnique CSEM SA, 2002 Neuchâtel, Switzerland

**Keywords:** uterine peristalsis, uterine contractility, MRI-cine, endometriosis, fertility, infertility

## Abstract

**Background:** Uterine contractility, also known as uterine peristalsis (UP), is a critical determinant of fertility, affecting sperm transport and embryo implantation. Increased uterine peristaltic activity has been associated with reduced pregnancy rates. However, data are heterogeneous and uterine contractility has not been widely translated into clinical practice. Cine-MRI, although limited by cost and heterogeneity in data reporting, has emerged as a promising tool to assess uterine dynamics and increase our knowledge of UP in physiological and pathological conditions. **Objective:** This systematic review and meta-analysis aimed to describe patterns of UP in physiological and pathological uterine conditions, including endometriosis and fibroids, using cine-MRI. **Methods:** A systematic literature search of the Medline, Embase, Cochrane and CENTRAL databases and Google Scholar was conducted up to May 2024, including studies evaluating UP by cine-MRI. Clinical studies evaluating uterine contractility were included, excluding those affected by therapeutic interventions or unrelated pathologies. This meta-analysis pooled data from studies comparing uterine contractility in patients with endometriosis. **Results:** In the 13 included studies (365 women), uterine contractility varied significantly according to menstrual cycle phases and pathological conditions. This meta-analysis showed that women with endometriosis had higher uterine contractility in the luteal phase (0.74; 95% CI: 0.27–1.21) but not in the periovulatory phase (SMD 0.8; 95% CI: −3.78–5.37). **Conclusions:** Cine-MRI is a promising diagnostic tool for the analysis of UP. Endometriosis is associated with impaired UP, which may be a cause of the decreased implantation rate and infertility in endometriosis. However, further research is needed to consolidate the effect of UP on implantation and fertility and to develop standardised and cost-effective tools to assess uterine contractility and tailor infertility treatment.

## 1. Introduction

Uterine peristalsis (UP) is a wave-like motion that arises in the uterine junctional zone continuously throughout the menstrual cycle in the non-pregnant uterus. It is involved in active sperm transport and embryo implantation of the embryo and is therefore an important factor in fertility [1]. As infertility becomes more prevalent, impaired uterine contractility caused by uterine pathologies such as fibroids and endometriosis may play a significant role in reduced fertility.

The junctional zone, also known as the stratum submucosum of the myometrium, is a thin layer adjacent to the endometrium that shares a common ontogenic origin, the paramesonephric duct [2,3]. The junctional zone consists mainly of longitudinal smooth muscle fibres and, under the influence of estrogen and progesterone, elicits contractions that vary in amplitude, frequency and direction throughout the menstrual cycle [3,4].

Although UP has been studied since the 1990s, the data are still heterogeneous and have hardly been translated into clinical practice. One of the reasons for this is that the tools used to analyse UP vary widely between studies, and no gold standard method has yet been established. Tools using intrauterine pressure catheters or electrodes for electrohysterography are not reliable enough as they may alter the characteristics of the peristalsis due to their invasive nature [5].

Vaginal ultrasound has been widely used due to its accessibility and advances in ultrasound technology, as well as analysis techniques involving visual or computerised evaluation of video clips. However, the use of UP in research and clinical settings is limited due to its subjectivity, interobserver variability and lack of consensus regarding the classification of observations [6,7,8].

Furthermore, even though pathologies such as endometriosis, adenomyosis and uterine fibroids have been associated with altered contractility of the junctional zone and infertility [1,9,10], the existing literature is still scarce and heterogenous.

Magnetic resonance imaging (MRI) using the cine-mode display has been used in recent studies to assess uterine contractility. Even though its cost and availability may limit its use [11,12,13,14,15], the objectivity and computerised analysis of MRI scans promise to increase our knowledge of the clinical relevance of UP in fertility and infertility. Furthermore, cine-MRI has demonstrated its potential as a valuable diagnostic tool for endometriosis, revealing increased uterine contractility during the luteal phase in affected women and providing dynamic information regarding the mobility and adherence of pelvic organs, which is essential for effective therapeutic planning.

We therefore systematically reviewed and conducted a meta-analysis of the existing literature to evaluate the use of cine-MRI as a measurement tool for assessing UP and its patterns under both physiological and pathological conditions. Our review specifically focused on the impact of uterine abnormalities, such as endometriosis and fibroids, on UP and their potential implications for fertility.

## 2. Materials and Methods

### 2.1. Registration of Protocols

The protocol was registered in the Prospective International Registry of Systematic Reviews, PROSPERO (registered number CRD 42024591834). The guidelines for the Preferred Reporting Items for Systematic Reviews and Meta-Analyses (PRISMA) have been used [16].

### 2.2. Search Strategy

A systematic literature search was conducted using the Medline, Embase, Cochrane and CENTRAL databases as well as Google Scholar in May 2024. A specialised librarian developed an initial Embase search strategy and tested a basic reference list. Following refining and querying, complex search strategies were developed for each information source based on database-specific controlled vocabularies (thesaurus terms/headings) and text words. Synonyms, acronyms and similar terms were included in the text word search. Publications from 1946 to the present were included in the search. Search terms included ‘uterine contractions’, ‘uterine peristalsis’, ‘junctional contractions’ and ‘cine magnetic resonance imaging’. Animal-only studies were excluded from all the database searches using a double-negative search strategy based on the Ovid ‘humans only’ filter. Duplicates were manually removed in EndNote [17]. The detailed final search strategies are provided in the Supplementary File S1. Reference lists and bibliographies of the relevant publications were screened for relevant studies in addition to the electronic database searches. All the identified citations were imported into Covidence.

### 2.3. Inclusion and Exclusion Criteria

The studies were independently assessed for inclusion using Covidence software (www.covidence.org) accessed on May 2024 [18] by the investigators AV and CB. Eligibility was determined on the basis of original publications that provided information on uterine contractions using cine-MRI. We excluded trials that used therapeutic interventions that affected contractility (such as various pharmacological treatments and others), trials with an inadequate design or trials that used animals.

### 2.4. Data Extraction

Two independent investigators (A.V. and C.B.) extracted data from the selected studies. Discrepancies were resolved through discussion and consensus. Data extraction focused on study characteristics (e.g., author, year, country and study design), patient demographics (age, cause and duration of infertility) and variables related to uterine contractility (e.g., time of measurement and number of contractions per minute). The data were obtained from the text, tables and figures, with additional calculations performed when required (Table 1).

### 2.5. Quality Assessment

The Newcastle–Ottawa scale (NOS) was utilised to evaluate the quality of the individual studies [19]. Three parameters were considered for individual study scoring: subject selection (0–4 stars), comparability (0–2 stars) and study outcome (0–3 stars). The scoring was composed as follows: good quality (=3 or 4 stars in the selection domain AND 1 or 2 stars in the comparability domain AND 2 or 3 stars in the outcome/exposure domain), fair quality (=2 stars in the selection domain AND 1 or 2 stars in the comparability domain AND 2 or 3 stars in the outcome/exposure domain) and poor quality (=0 or 1 star in the selection domain OR 0 stars in the comparability domain OR 0 or 1 stars in the outcome/exposure domain). All the included studies were reviewed by AV and CB to independently assess the risk of bias. Disagreements were resolved by consensus. Scoring was conducted according to the terms listed in Table 2.

### 2.6. Data Synthesis

The primary outcome was the assessment of UP in cine-MRI. For this meta-analysis, we analysed a subgroup of patients consisting of women with and without endometriosis. We used the mean and standard deviation (SD) of the frequency of uterine contractions per 2 min in both the endometriosis group and the control group during the periovulatory and luteal phases, as reported in each study. The pooled results of the standardised mean differences (SMDs) with 95% CIs in levels of UP were analysed using the “metafor” and “dmetar” functions in R software ((Version 4.2.3, R Core Team, Vienna, Austria, 2013). To examine heterogeneity, we used Cohen’s Q statistic and I2 statistic. In the presence of high heterogeneity, we employed random-effects models.

## 3. Results

### 3.1. Results of the Systematic Review

**Figure 1:**
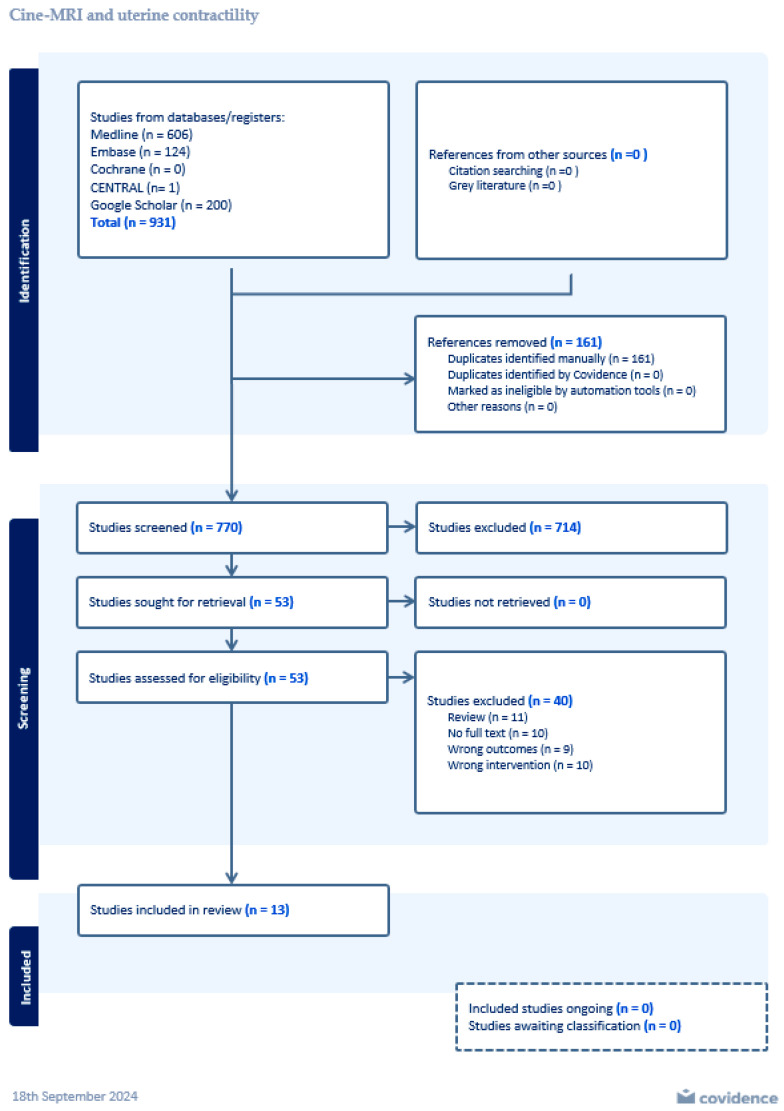
PRISMA flow chart. Flow chart of the bibliography search and selection process.

After screening the abstracts and the full text of the study topic, 53 studies remained. However, we excluded 40 of these studies as they did not fit our predetermined inclusion criteria. Therefore, we included 13 articles in the systematic review (Figure 1).

### 3.2. Study Characteristics

The characteristics of the study populations are summarised in Table 1. Sixteen studies were prospective. The patients included in the 13 studies were predominantly Asian (9 studies), European (1 study) and American (3 studies). A total of 365 women were included in the review. Eighty-one women (22.2%) were eligible for subgroup analysis, which was the basis for this meta-analysis. The study sample sizes varied from 7 to 55 patients. The methodological quality of the studies was rated as either good for seven of them [20,21,22,23,24,25,26] or poor [27,28,29,30,31,32] (n = 6), primarily due to the absence of a comparison group (Table 2).

### 3.3. Results of Individual Studies

Cine-MRI measurements differ in duration depending on the study, with some using short and some using long imaging sessions focused on specific phases of the menstrual cycle.

1. Frequency of uterine contractions

Depending on the phase of the menstrual cycle and the presence of underlying pathological conditions, the frequency of uterine contractions showed significant variability. Maximum contraction frequency during the periovulatory phase is 4.4 min in healthy controls, whereas PCOS patients show less UP [22]. Uterine fibroids are associated with reduced contraction frequency [23].

2. Amplitude of uterine contractions

Contraction amplitude measurements indicate weaker uterine contractions under certain conditions. Breast-feeding women have significantly reduced contraction amplitudes (mean 2.8 ± 1.9 mmHg) compared to controls (mean 6.3 ± 1.8 mmHg), suggesting reduced endometrial remodelling required for implantation [24]. In addition, women with fibroids have reduced contraction amplitudes and frequencies compared to healthy women [23,29,33].

3. Direction of uterine contractions

Depending on the phase of the cycle, the direction of uterine contractions varies. Cervix-to-fundus contractions facilitate sperm transport and implantation [4,20]. Altered contraction patterns, including prolonged and atypically directed contractions, may affect reproductive processes [21].

4. Uterus contractility in various physiological conditions or pathologies

*Lactation:* A decreased uterine contractility, both in frequency and amplitude, is associated with breast feeding, as observed by Daido et al. (2016). A reduction in uterine contractility may have an effect on implantation and reproductive outcomes [24].

*Polycystic Ovarian Syndrome (PCOS):* PCOS affects UP, reducing the frequency and activity of contractions, especially during the periovulatory phase, and therefore may affect the success of implantation [22].

*Uterine fibroids:* Uterine fibroids, particularly intramural types, are associated with a significant reduction in the frequency and amplitude of uterine contractions when compared to controls. Such alterations of physiological anatomy that also cause the disruption of UP suggest that they may adversely affect embryo implantation [23].

*Endometriosis*: Endometriosis patients have significantly altered contraction frequencies and abnormal patterns, particularly in the periovulatory phase [21,26].

### 3.4. Meta-Analysis Results

#### Uterine Contractility in the Endometriosis Group

Two studies [21,26] were included, comparing the frequency of uterine contractions during the periovulatory and luteal phases in women with endometriosis (44 women) and without endometriosis (37 women). For cases of endometriosis, both deep endometriosis and peritoneal endometriosis were considered.

Mean uterine contractility (luteal phase) was significantly higher in women with endometriosis compared to women without endometriosis, resulting in a positive standardised mean difference (SMD) (SMD = 0.74, 95% CI: 0.27; 1.21 and *p* < 0.001). No heterogeneity was observed between studies (I² = 0%; *p* < 0.94). In contrast, no significant difference in uterine contractility was evident in the periovulatory phase (SMD = 0.8; 95% CI: 3.78–5.37). There was substantial heterogeneity between the studies (I^2^ = 98%; *p* < 0.01).

**Figure 2:**
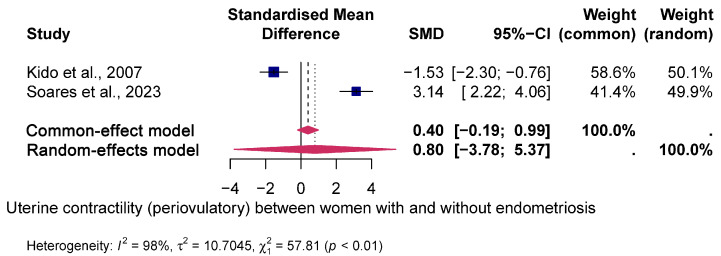
Pooled global uterine contractility (periovulatory) [21,26] between women with and without endometriosis. Forest plot of odds ratios (ORs) and 95% confidence intervals (CIs) for studies evaluating UP in women with and without endometriosis in the periovulatory phase. The blue squares in each study indicate the OR, the size of the squares indicates the study weight and the horizontal lines indicate the 95% CI. The data in the blue diamond represent the pooled OR in patients with high vs. low uterine contractility and 95% CI. The overall estimates are presented in the fixed- and random-effects models. The prediction interval is defined as the interval within which the effect size of a new study would fall if this study were selected at random from the same population of studies already included in this meta-analysis.

**Figure 3:**
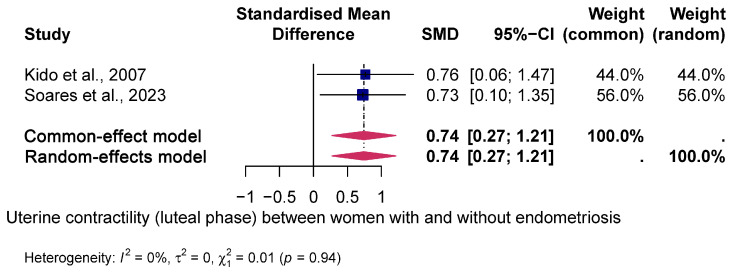
Pooled global uterine contractility (luteal phase) [21,26] between women with and without endometriosis. Forest plot of odds ratios (ORs) and 95% confidence intervals (CIs) for studies evaluating UP in women with and without endometriosis in the luteal phase. The blue squares in each study indicate the OR, the size of the squares indicates the study weight and the horizontal lines indicate the 95% CI. The data in the blue diamond represent the pooled OR in patients with high vs. low uterine contractility and 95% CI. The overall estimates are presented in fixed- and random-effects models. The prediction interval is defined as the interval within which the effect size of a new study would fall if this study were selected at random from the same population of studies already included in this meta-analysis.

Figure 2 and Figure 3 show the forest plot of the pooled SMD between both groups.

## 4. Discussion

The aim of this systematic review was to analyse the existing data on UP and its patterns across various uterine pathologies, with cine-MRI as the measurement tool. This first meta-analysis on UP in women with and without endometriosis diagnosed via cine-MRI reveals increased luteal-phase contractility in the endometriosis group, emphasising cine-MRI’s role in detecting pathological peristalsis patterns.

In this review, we considered seven good-quality prospective studies [20,21,22,23,24,25,26] and highlighted some key findings. First, the mean uterine contractility was significantly higher in women with endometriosis compared to women without endometriosis in the luteal phase; however, there was no difference in UP in the periovulatory phase (see Figure 2 and Figure 3). Second, the mean uterine contractility was significantly higher in women with endometriosis compared to women without endometriosis in the luteal phase; however, there was no difference in UP in the periovulatory phase.

The contractile activity of the uterus during the natural menstrual cycle has been described to vary in frequency, amplitude and direction (Table 3). Prior to ovulation, an increased frequency of uterine contractions in the cervico-fundal direction is observed, facilitating the transport of spermatozoa to the fallopian tubes. Such changes in the direction of the wave-like movements are caused by the production of local hormones in the dominant follicle [34]. During the luteal phase, the uterus undergoes hypokinetic changes, creating a suitable environment for embryo implantation [35,36]. These findings support the hypothesis that uterine contractility activity plays a central role in fertility and that gynaecological pathologies affect the quality and quantity of these contractions [3,6].

Endometriosis affects approximately 5–10% of the reproductive age population and is one of the most common gynaecological disorders. Endometriosis has a significant impact on fertility and quality of life [4,37,38]. It represents a broad spectrum of pathologies, ranging from ovarian endometriosis, as analysed by Kido et al., 2007, to deep infiltrating endometriosis as analysed by Soares et al., 2023. Furthermore, endometriosis is frequently associated with adenomyosis, a closely related disorder with very similar pathophysiology [39,40]. Accordingly, all these entities have abnormal UP as shown by several studies.

Uterine contractility is abnormal in endometriosis, which may facilitate its development through retrograde menstruation [3,41]. Furthermore, abnormal uterine contractility is thought to be a mechanical factor contributing to infertility [10]. Our meta-analysis results emphasise the significance of abnormal uterine contractility activity and its association with endometriosis as we found an increased frequency of uterine contractions during the luteal phase in women with endometriosis.

In line with the similarities in pathophysiology between different types of endometriosis and adenomyosis, UP is also abnormal across the broad spectrum of endometriosis and adenomyosis. Arena et al., 2024, analysed uterine contractility by ultrasound in 18 women with adenomyosis during all phases of the menstrual cycle and compared the contractility with healthy controls. They also found abnormal contractility patterns in terms of amplitude, frequency, velocity and dynamics [42]. Soares et al., 2007, analysed uterine contractility using cine-MRI in 18 women with deep infiltrating endometriosis and also found disturbed UP, which could have a negative effect on fertility, particularly sperm transport and implantation. Cine-MRI represents a valuable diagnostic tool for endometriosis, providing dynamic information regarding the mobility and adherence of pelvic organs, which is essential for effective therapeutic planning Supplementary File S2.

UP has been studied extensively over the years, but a definitive and widely accepted method of measuring uterine contractions has not been established due to the inherent limitations of all the currently available diagnostic tools. Traditional methods such as intrauterine pressure (IUP) measurement are suboptimal—the invasive nature can potentially alter the characteristics of UP. Although transvaginal ultrasound, particularly 2D modalities, is widely used, it is highly operator-dependent and subjective, posing significant challenges for standardisation. Advances in 3D and 4D modes of transvaginal ultrasound have improved imaging capabilities but still require a high level of expertise.

However, the logistical challenges and operator dependency of these diagnostics limit their use alongside fertility treatment. Such limitations open the door to newer techniques such as cine-MRI, as analysed in this review, but also electrohysterography (EHG), which is a promising tool for objective, non-invasive measurement. In addition to providing data on uterine activity, EHG avoids the practical limitations of imaging techniques and offers greater reproducibility and integration into clinical practice.

Future research should focus on validating such tools for clinical use and exploring their potential to accurately measure UP. In addition, future clinical trials are needed to standardise not only the procedure but also the interpretation of the results. These advances could greatly improve the personalisation of infertility and endometriosis treatment. The results of our systematic review indicate that cine-MRI is an effective tool for assessing and understanding uterine dynamics, which is valuable for diagnosis and possible treatment planning. The potential investigation of altered uterine contractility in women with endometriosis and possible interventional treatments to modulate this contractility should also be explored.

## 5. Study Limitations

Our review has some limitations, despite strictly following the recommendations for high-quality evidence summaries. Firstly, the sample sizes in the trials of certain clinical conditions were very limited, so it was not possible to perform subanalyses, such as focusing on women with PCOS or with fibroids. This meta-analysis only included two trials, which limits its power. Secondly, most of the included trials were based on very heterogeneous populations. Finally, the data on parameters of uterine activity were inconsistent or poorly described.

## 6. Conclusions

In conclusion, cine-MRI is a promising diagnostic tool for the analysis of UP. Endometriosis is associated with disturbed UP, which may be a cause of the decreased implantation rate and infertility in endometriosis. Additionally, the findings indicate that specific endometriosis phenotypes may predispose individuals to reduced uterine peristalsis, warranting deeper exploration of the underlying mechanisms and their potential clinical implication. However, further research is needed to consolidate the effect of UP on implantation and fertility and to develop standardised and cost-effective tools to assess uterine contractility and tailor infertility treatments.

## Figures and Tables

**Table 1:** Characteristics of the included studies.

					Moment of Measure of Contractility (per Patient)				Frequency (Contractions/min)			Direction
Author, Year	Study Design	Number of Participants of Interest	Number of Controls	Age (yrs), Study Group	Follicular Phase	Periovulatory Phase	Luteal Phase	Menstrual Phase	Study Group	Controls	(*p* Value)	
Nishino at al., 2005 [27]	prospective	26	-	41 [19–51]	5	3	14	3	-		-	CF midcycle/FCl during MP
Kido at al., 2006 [20]	prospective	12	-	26.1 [22–31]	-	-	-	-	UP was identified in 93.8% of the studies during the POV, in 52.1% of the studies during the MP and in 31.3% of the studies during the LF		-	-
Kido at al., 2007 [21]	prospective case-control	26	12	35.1 [24–51]	-	10	13	3	2.5 ± 1/(2 min)	4.4 ± 1.6/2 min	<0.05	CF
Orisaka et al., 2007 [28]	prospective	19	3	34.8 [24–42]	2	1	11	5	-		-	-
Yoshino et al., 2010 [29]	prospective	51	-	36.5 [29–41]	-	-	51	-	-	-	-	-
Leonhardt et al., 2012 [22]	prospective case-control	55	28	29.5 +/− 4.5	6	14	7	-	1.5 ± 0.6	1.1 ± 0.5	0.01	CF
Yoshino et al., 2012 [30]	prospective	15	-	-	-	-	15	-	2−6	-	-	-
Kido et al., 2014 [23]	prospective case-control	20	20	45.5 +/− 3.7	-	40	-	-	1.3−2.5	7.6−4.7	<0.05	CF
Shitano et. al, 2015 [31]	prospective	31	-	30.0 [20–44]	0	31	0	0	4.4−5.1	-	-	-
Daido et al., 2016 [24]	prospective case-control	22	16	29.0 +/− 3.2	13	-	12	-	2.8 ± 1.9	6.3 ± 1.8	<0.01	-
Kiguchi et al., 2017 [25]	prospective case-control	28	50	62.6 [55–71]	-	28	-	-	0	4.5−4.6	<0.0001	-
Nakashima et al., 2019 [32]	prospective	7	-	40 ± 2	4	2	6	7	max 6.5 ± 0.5 (no time unit)		-	CF/bilateral cornua to cervix and opposite cornu
Soares at al., 2023 [26]	prospective case-control	18	25	36.6 ± 6.0	6	15	22	0	3.83 ± 0.48/(2 min)	2.44 ± 0.4	0.23	CF or FC

**Abbreviations:** LP: luteal phase, POV: periovulatory phase, MP: menstrual phase, CF: cervix to fundus, FC: fundus to cervix, yrs: years and min: minutes.

**Table 2:** Newcastle–Ottawa quality assessment form for cohort studies.

First Author, Year of Publication	Representativeness of Exposed Cohort	Selection of Non-Exposed Cohort	Ascertainment of Exposure	Outcome of Interest Not Present at Study Start	Comparability of Cohorts on the Basis of the Design or Analysis Controlled for Confounders	Assessment of Outcome	Sufficient Length of Follow-Up for Outcomes to Occur	Adequacy of Follow-Up of Cohorts	Total	Quality Assessment
Nishino at al., 2005 [27]	★	-	★	★	-	★	★	★	6	Poor quality.
Kido et al., 2006 [20]	★	-	★	★	-	★	★	★	6	Good quality.
Kido et al., 2007 [21]	★	★	★	★	★	★	★	★	8	Good quality.
Orisaka et al., 2007 [28]	★	-	★	★	-	★	★	★	6	Poor quality.
Yoshino et al., 2010 [29]	★	-	★	★	-	★	★	★	6	Poor quality.
Leonhardt et al., 2012 [22]	★	★	★	★	★	★	★	★	8	Good quality.
Yoshino et al., 2012 [30]	★	-	★	★	-	★	★	★	6	Poor quality.
Kido et al., 2014 [23]	★	★	★	★	★	★	★	★	8	Good quality.
Shitano et. al, 2015 [31]	★	-	★	★	-	★	★	★	6	Poor quality.
Daido et al., 2016 [24]	★	★	★	★	★	★	★	★	8	Good quality.
Kiguchi et al., 2017 [25]	★	★	★	★	★	★	★	★	8	Good quality.
Nakashima et al., 2019 [32]	★	-	★	★	-	★	★	★	6	Poor quality.
Soares et al., 2023 [26]	★	★	★	★	★	★	★	★	8	Good quality.

**Table 3:** Characteristics of UP. Frequency, direction and amplitude of UC in different phases of the menstrual cycle.

Phase	Subphase	Frequency (Contractions/min)	Amplitude (mmHg)	Direction
Menstruation		0.33–3.0	13.6	FC
Follicular phase	Mild	1.5–3.3	5.2	FC/CF
	Late	3.0–6.0	2.9	CF
Luteal phase	Early	2.0–4.0	Amplitude rises	
	Late	0.8–1.8	CF/opposing	Opposing/no contractions

The frequency, direction and amplitude of uterine contractions at different phases of the menstrual cycle (van Gestel et al., 2003 [7]). Abbreviations: CF = cervix to fundus; FC = fundus to cervix; opposing = contractions are initiated in both the cervical and fundal regions; UP = uterine peristalsis; and UC = uterine contraction.

## Data Availability

The current study was based on results of relevant published studies.

## Supplementary Materials

The following supporting information can be downloaded at https://www.mdpi.com/article/10.3390/jcm14031021/s1. Supplementary File S1: Database searching strategies systematic literature search in Medline, Embase, Cochrane, CENTRAL and Google Scholar. Supplementary File S2: Video of uterine contractility, assessed by cine-MRI.
