# Supplementary material for: Cine-MRI for Quantifying Uterine Peristalsis: A Systematic Review and Meta-Analysis
Source: J Clin Med. 2025 Feb 6;14(3):1021. doi: 10.3390/jcm14031021 (PMC11818271; doi:10.3390/jcm14031021)
Supplement: Supplementary file 1 [file jcm-14-01021-s001.zip › jcm-3437729-Supplementary File S1.pdf]

Supplement: Cine MRI and uterine contractility

Detailed search strategy

To identify potentially relevant publications on the topic, a search strategy was designed, and investigated in Embase, MEDINE, Cochrane Database of Systematic Review and Cochrane CENTRAL Register of Controlled Trials. The core concepts of the search strategy consisted of *cine mri* and *uterine contractility*. A medical information specialist developed an initial search strategy in Embase and tested it against a list of core references to ensure key publications were included. After refinement, the information specialist set up the search strategy for each information source based on database-specific index terms and free text. The free text search included synonyms, acronyms, and similar terms. Database provided language limits have been applied for English, German, French, Italian, Spanish and Portuguese articles. No other limits have been applied. Studies concerning exclusively animals were excluded from the searches by using a double-negative search strategy based on the "Humans only" filters by Ovid. In addition, a keyword search was carried out in Google Scholar using the Publish or Perish software[1]. The 200 references displayed first (relevance ranking) were integrated. The search was performed on May 29, 2024. The results were imported to EndNote and deduplicated following the “Bramer Method”[2]. Further processing of the records took place in Covidence.

Search details

**Database searched**	**Platform**	**Years of coverage**	**Date of search**	**Records**	**Records after deduplication**
Medline ALL	Ovid	1946-2024 May 28	2024 May 29	124	124
Embase	Ovid	1974-2024 May 28	2024 May 29	606	491
Cochrane Database of Systematic Reviews	Wiley	1992 to present	2024 May 29	0	0
Cochrane CENTRAL Register of Controlled Trials	Wiley	1992 to present	2024 May 29	1	0
Additional Search Engine: Google Scholar				200	155
**Total**				**931**	**770**

Search Strategies

****

Ovid MEDLINE(R) ALL <1946 to May 28, 2024>
Search date: May 29, 2024

1 magnetic resonance imaging/ or exp magnetic resonance imaging, cine/ 497633

2 ((mri or mr or magnetic resonance or fmri) adj4 (cine or serial or sequential* or ultrafast or dynamic or real-time or functional or motion or live)).ti,ab,kw. 84227

3 or/1-2 521623

4 exp Uterine Contraction/ 7892

[1] Harzing, A.W. (2007) Publish or Perish, available from https://harzing.com/resources/publish-or-perish
[2] Bramer WM, Giustini D, de Jonge GB, Holland L, Bekhuis T. De-duplication of database search results for systematic reviews in EndNote. J Med Libr Assoc. 2016 Jul;104(3):240-3. doi: 10.3163/1536-5050.104.3.014. Erratum in: J Med Libr Assoc. 2017 Jan;105(1):111. PMID: 27366130; PMCID: PMC4915647.

5 exp Peristalsis/ and exp Uterus/44

6 ((Uterine or uterus or myometri* or womb) adj4 (contracti* or peristal* or motil* or activit* or function* or wave*1)).ti,ab,kw. 15191

7 or/4-6 18453

8 3 and 7 130

9 (exp animal/ or exp invertebrate/ or animal experiment/ or animal model/ or exp plant/ or exp fungus/) not exp human/ 5666933

10 8 not 9 127

11 limit 10 to (english or french or german or italian or portuguese or spanish) 124

****

Embase <1974 to 2024 May 28>
Search date: May 29, 2024

1 nuclear magnetic resonance imaging/ or exp cine magnetic resonance imaging/ 1058130

2 ((mri or mr or magnetic resonance or fmri) adj4 (cine or serial or sequential* or ultrafast or dynamic or real-time or functional or motion or live)).ti,ab,kw. 111870

3 or/1-2 1120877

4 exp uterus contractility/ 3543

5 exp uterus contraction/ 11624

6 uterine peristalsis.dj. or (exp uterus/ and peristalsis/) 182

7 ((Uterine or uterus or myometri* or womb) adj4 (contracti* or peristal* or motil* or activit* or function* or wave*1)).ti,ab,kw. 17736

8 or/4-7 24139

9 and/1,8623

10 (exp animal/ or exp invertebrate/ or nonhuman/ or animal experiment/ or animal tissue/ or animal model/ or exp plant/ or exp fungus/) not (exp human/ or human tissue/) 7933957

11 9 not 10 613

12 limit 11 to (english or french or german or italian or portuguese or spanish) 606

****

Cochrane Database <1992-present>
Search date: May 29, 2024

#1 [mh ^"magnetic resonance imaging"] OR [mh "magnetic resonance imaging, cine"] 11751

#2 ((mri OR mr OR "magnetic resonance" OR fmri) NEAR/4 (cine OR serial OR sequential* OR ultrafast OR dynamic OR real-time OR functional OR motion OR live)):ti,ab,kw 9546

#3 #1 OR #2 17813

#4 [mh "Uterine Contraction"] 465

#5 [mh Peristalsis] AND [mh Uterus] 0

#6 ((Uterine OR uterus OR myometri* OR womb) NEAR/4 (contracti* OR peristal* OR motil* OR activit* OR function* OR wave*)):ti,ab,kw 2769

#7 #4 OR #5 OR #6 2769

#8 #3 AND #7 1

#9 ([mh animal] OR [mh invertebrate] OR [mh ^"animal experiment"] OR [mh ^"animal model"] OR [mh plant] OR [mh fungus]) NOT [mh human] 3869

#10 #8 NOT #9 1

****

Google Scholar
Search date: May 29, 2024

(“cine mri”|”cine magnetic resonance”) AND (“uterine contractility”|”uterus contraction”|”uterine peristalsis”)

Filters: Any time - Sort by relevance any scholarly work type - exclude patents -exclude citations
